# Supplementary material for: How Interdisciplinary Interventions Can Improve the Educational Process of Children Regarding the Nutritional Labeling of Foods
Source: Foods. 2023 Nov 28;12(23):4290. doi: 10.3390/foods12234290 (PMC10706482; doi:10.3390/foods12234290)
Supplement: Supplementary file 1 [file foods-12-04290-s001.zip › foods-2434129-supplementary.pdf]

Questionnaire: Behaviors, knowledge, and preferences about nutrition labeling

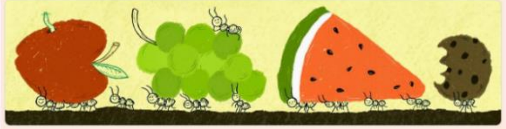

BEHAVIOR, KNOWLEDGE AND PREFERENCES ABOUT NUTRITION LABELING

You will now take part in a survey to assess your attitudes, knowledge, and preferences about the nutrition label on food product packaging

a) Behaviors and knowledge about nutrition labeling

1. The nutrition label should be present on all food product packaging. Do you know what it means? *
   - ○ Yes
   - ○ No
   - ○ Maybe

2. Do you usually look at the nutrition label on food packages? *
   - ○ Yes
   - ○ No
   - ○ Sometimes

3. Do you find it easy to find the nutrition label on food packages? *
   - ○ Yes
   - ○ No
   - ○ Sometimes

4. Do you find it easy to understand the nutrition label information on food packages? *
   - ○ Yes
   - ○ No
   - ○ Sometimes

5. Can you tell if a food product is healthy or not just by looking at the nutrition label? *
   - ○ Yes
   - ○ No
   - ○ Maybe / Sometimes

6. Do you think it is important for a food product to have the nutrition label on the package? *
   - ○ Yes
   - ○ No
   - ○ Maybe

7. Do you know why a food product needs to have the nutrition label on the package? *
   - ○ Yes
   - ○ No
   - ○ Maybe

8. Do you pay attention to the nutrients on the nutrition label on food product packaging? *
   - ○ Yes
   - ○ No
   - ○ Sometimes

9. Do you think that foods rich in sugar, fat and sodium are bad for your health? *
   - ○ Yes
   - ○ No
   - ○ Sometimes

10. Do you find it easy to understand the list of ingredients present on food product packaging? *
   - ○ Yes
   - ○ No
   - ○ Sometimes

11. Look at the nutrition label below (TABLE) and indicate in which location the nutrition label should appear on food product packaging: *

Nutrition Facts<br>Serving Size: 7oz. (200ml)<br>Serving Per Container 1		
Amount Per Serving		% Daily Value
Calories	132	7%
Carbohydrate	22g	7%
Protein	7g	9%
Total Fat	5,5g	10%
Saturaded Fat	4g	18%
trans *Fat*	0g	
Dietary Fiber	2g	8%
Sodium	117mg	5%
* Percent Daily Value are based on a 2.000 calorie diet.		
Ingredients: Whole reconstituted milk, milk permeate, whey concentrate, cocoa syrup (water and cocoa), liquid sugar, Vitamins (C, B3, E, B6, B2, A), salt, flavoring agents, stabilizers (disodium phosphate, guar gum, carrageenan and citric acid acidulant).		

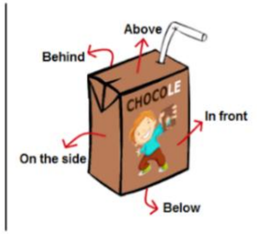

   - ☐ Behind
   - ☐ Above
   - ☐ On the side
   - ☐ In front
   - ☐ Below
   - ☐ Don't know

12. Look at the nutrition label below (TRAFFIC LIGHT) and indicate in which location the nutrition label should appear on food product packaging: *

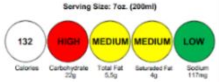

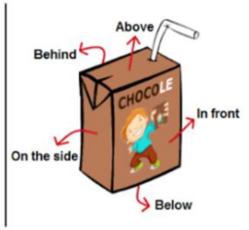

   - ○ Behind
   - ○ Above
   - ○ On the side
   - ○ In front
   - ○ Below
   - ○ Don't know

13. Look at the nutrition label below (ALERT) and indicate in which location the nutrition label should appear on food product packaging: *

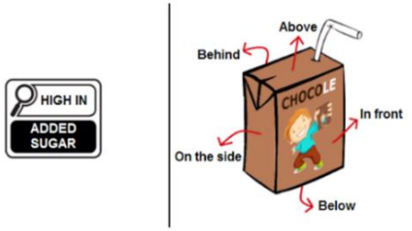

   - ○ Behind
   - ○ Above
   - ○ On the side
   - ○ In front
   - ○ Below
   - ○ Don't know

14. If you notice that the food product has nutrients that are bad for your health, what do you do? *
   - ○ Eats anyway
   - ○ Doesn't eat
   - ○ Don't know

15. Do you think it is important to look at the ingredients list of a food product (what it is made of)? *
   - ○ Yes
   - ○ No
   - ○ Maybe / Sometimes

16. If you checked YES in the previous question (15), why do you think it is important to look at the ingredients list of a food product?
   - ○ To see if it is healthy
   - ○ To see if it is bad for your health
   - ○ Other - describe
   - ○ ____________________

b) Preferences on nutrition labeling

17. Take a look at the image below. Do you think it is cooler for the nutrition label * to appear in which place on the package?

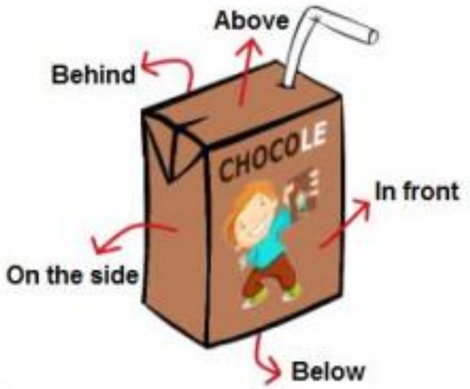

- ◯ Behind
- ◯ Above
- ◯ On the side
- ◯ In front
- ◯ Below
- ◯ Don't know

18. Which of these three nutrition labels do you think is coolest to appear on the * package?

1) Table

Nutrition Facts		
Serving Size: 7oz. (200ml)<br>Serving Per Container 1		
Amount Per Serving		% Daily Value
Calories	132	7%
Carbohydrate	22g	7%
Protein	7g	9%
Total Fat	5,5g	10%
Saturaded Fat	4g	18%
trans *Fat*	0g	
Dietary Fiber	2g	8%
Sodium	117mg	5%
* Percent Daily Value are based on a 2.000 calorie diet.		
Ingredients: Whole reconstituted milk, milk permeate, whey concentrate, cocoa syrup (water and cocoa), liquid sugar, Vitamins (C, B3, E, B6, B2, A), salt, flavoring agents, stabilizers (disodium phosphate, guar gum, carrageenan and citric acid acidulant).		

2) Traffic Light

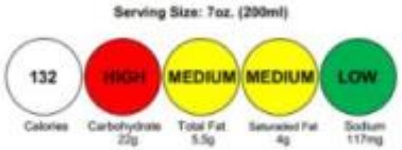

3) Alert

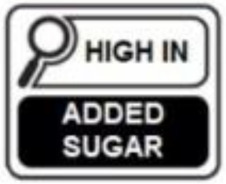

- ◯ 1) TABLE
- ◯ 2) TRAFFIC LIGHT
- ◯ 3) ALERT

Questionnaire: Perception of healthiness among different nutrition labels

PERCEPTION OF HEALTHINESS AMONG DIFFERENT NUTRITION LABELS

In the pictures below, you will see different categories of labels for FILLED SWEET BISCUITS. For each category, evaluate the labels presented and mark what you think of the food that presents that label: 1. Not healthy; 2. Unhealthy; 3. Neither healthy nor unhealthy; 4. Healthy; 5. Very healthy.

Nutrition labels of stuffed sweet cookies in TABLE form *

ROOT A

Nutrition Facts

Serving Size: 30g
Serving Per Container 3

Amount Per Serving		% Daily Value
Calories	130	7%
Carbohydrate	21g	7%
Protein	2g	3%
Total Fat	3,1g	6%
Saturaded Fat	0,5g	2%
trans *Fat*	0g	
Dietary Fiber	1g	4%
Sodium	42mg	2%

* Percent Daily Value are based on a 2.000 calorie diet.

Ingredients: Whole Wheat Flour, Invert Sugar, Vegetable Fat, Modified Starch, Cocoa Powder, Salt, Breadcrumbs, Oat Flour, Whey Permeate, Whole Milk Powder, Vitamins: B1, B2, B3, B6 and A, chemical yeasts (ammonium bicarbonate, monocalcium phosphate and sodium bicarbonate), emulsifier (and sorbitan tristearate) and flavoring.

ROOT B

Nutrition Facts

Serving Size: 30g
Serving Per Container 3

Amount Per Serving		% Daily Value
Calories	125	6%
Carbohydrate	18g	6%
Protein	1,9g	3%
Total Fat	5,5g	10%
Saturaded Fat	3,2g	15%
trans *Fat*	0g	**
Dietary Fiber	0,8g	3%
Sodium	51mg	2%

* Percent Daily Value are based on a 2.000 calorie diet.

Ingredients: Wheat flour enriched with iron and folic acid, sugar, vegetable oil, invert sugar, rye flour, cocoa, calcium carbonate, whey, cocoa liquor, powdered milk, starch, salt, Vitamins: B1, B2, B3, B6 and A, emulsifiers (soy lecithin and sorbitan tristearate), chemical yeasts (monocalcium phosphate and sodium bicarbonate) and flavorings.

	1. Not healthy	2. Unhealthy	3. Neither healthy nor unhealthy	4. Healthy	5. Very healthy
Evaluating ROOT A, you think the cookie is:	☐	☐	☐	☐	☐
Evaluating ROOT B, you think the cookie is:	☐	☐	☐	☐	☐

Nutrition labels of stuffed sweet cookies in TRAFFIC LIGHT form. *

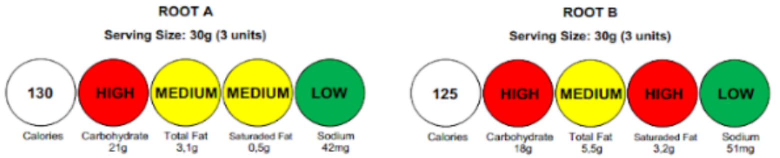

	1. Not healthy	2. Unhealthy	3. Neither healthy nor unhealthy	4. Healthy	5. Very healthy
Evaluating ROOT A, you think the cookie is:	☐	☐	☐	☐	☐
Evaluating ROOT B, you think the cookie is:	☐	☐	☐	☐	☐

Nutrition labels of stuffed sweet cookies in ALERT form. *

ROOT A **ROOT B**

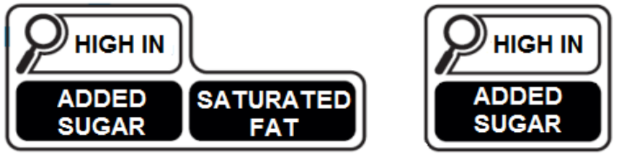

	1. Not healthy	2. Unhealthy	3. Neither healthy nor unhealthy	4. Healthy	5. Very healthy
Evaluating ROOT A, you think the cookie is:	☐	☐	☐	☐	☐
Evaluating ROOT B, you think the cookie is:	☐	☐	☐	☐	☐
